# Effectiveness comparisons of Chinese patent medicine on insomnia: A protocol for systematic review and Bayesian network meta-analysis

**DOI:** 10.1097/MD.0000000000024446

**Published:** 2021-02-05

**Authors:** Ruoyu Chao, Chunli Wu, Hongqiang An, Bing Li, Jianlin Wu

**Affiliations:** a College of Traditional Chinese Medicine, Shandong University of Traditional Chinese Medicine, Jinan; b Xintai Affiliated Hospital of Shandong First Medical University, Taian, Shandong Province, PR China.

**Keywords:** Chinese patent medicine, insomnia, network meta-analysis, protocol

## Abstract

**Background:**

In recent years, the incidence of insomnia is increasing. However, the existing therapy methods for cannot fundamentally treat the disease. Meanwhile, Chinese patent medicine (CPM) plays an active role in the treatment of insomnia. However, there is no comparison and ranking of the efficacy of every CPM. Therefore, our study will use network meta-analysis to compare the efficacy of different CPM on insomnia, in order to provide evidence-based medical evidence for clinical treatment.

**Methods:**

We will search CNKI, Wanfang, VIP, CBM, Pubmed, Cochrane Library, Embase for the randomized controlled trials of CPM in the treatment of insomnia (up to December 31, 2020). We will use RevMan5.3, Stata15.1 and ADDIS software for statistical analysis. We will draw the surface under cumulative ranking area to predict the order of efficacy.

**Results:**

We aim to rank the efficacy and safety of different CPM for the treatment of insomnia.

**Conclusion:**

CPM plays a positive role in the treatment of insomnia and can provide evidence support for clinicians and patients

**INPLASY registration number:**

INPLASY2020120121

## 1 Introduction

Sleep is an indispensable and important physiological phenomenon for everyone. Nearly 1/3 of a person's life is spent on sleep, so as to ensure the normal and stable physiological functions of the body. The quality of sleep will directly affect the health of the body, the orderly progress of daily life and work, and the good development of interpersonal relations.^[1,2]^ However, with the rapid development of society, people's life rhythm is accelerating, and positively related to it is that people's life pressure is also increasing, and more and more people suffer from insomnia. Insomnia is a common disease in the world, occurring in all age groups. In the United States, insomnia affects about 1/3 of the population.^[3]^ Insomnia can cause a variety of health problems, such as cognitive function decline, emotional disorders, attention loss, decreased quality of life, and so on.^[4]^ Long-term insomnia leads to an increased risk of hypertension and myocardial infarction, as well as depression, obesity and metabolic abnormalities.^[5–7]^ In the drug treatment of insomnia, sedative hypnotics are mainly used to treat insomnia. Traditional drugs include barbiturates, benzodiazepines, and non-diazepines.^[8]^ In addition, antihistamines and melatonin receptor agonists have also been used in the treatment of insomnia in recent years, but there are many adverse reactions of these drugs, and they are easy to recur after withdrawal, and have some problems such as addiction, tolerance, or withdrawal reaction.

Traditional Chinese medicine has a long history of treating insomnia and has certain advantages, which can not only improve sleep quality, but also has fewer side effects and high safety. Studies have proved that Chinese patent medicine (CPM) are effective in treating insomnia.^[9–13]^ CPM is a preparation produced with Chinese medicinal materials as raw materials under the guidance of Chinese medicine theory and in accordance with prescribed prescriptions, production processes and quality standards. It is a traditional Chinese medicine preparation that has been repeatedly used in clinical practice, proven to be safe and effective, and has a fixed dosage form, including traditional dosage forms such as pills, powders, ointments, and pills, and modern dosage forms such as tablets, capsules, oral liquids, granules, soft capsules, and dripping pills. There are many types of CPM for the treatment of insomnia, with different emphasis on efficacy, and there is still a lack of direct comparative research between different CPM. It is impossible to evaluate the effectiveness of various CPM. Although some studies have used traditional meta-analysis methods to discuss, but traditional meta-analysis can only be used for pairwise comparison, it does not involve the comparison between multiple treatment measures. Network meta-analysis (NMA) is an extension of traditional meta-analysis whose advantage is to evaluate the efficacy of multiple interventions. Therefore, this study will use the method of NMA to compare the efficacy of different CPM on insomnia, and rank the intervention measures according to the efficacy, in order to provide evidence-based medical evidence for clinical treatment plans.

## 2 Methods

We will report the study strictly according to the Preferred Reporting Items for Systematic Reviews and Meta-Analysis Protocols.^[14]^

### 2.1 Study registration

We have registered this study on the International Platform of Registered Systematic Review and Meta-analysis Protocols with the registration number of INPLASY2020120121 (URL = https://inplasy.com/inplasy-2020-12-0121/)

### 2.2 Inclusion criteria

#### 2.2.1 Type of study

We will include randomized controlled trials (RCTs) with complete case data, whether blind or not, regardless of country or region, and the language will be limited to Chinese and English.

#### 2.2.2 Participants

According to the diagnostic criteria for insomnia promulgated by European Sleep Research Society,^[8]^ we will include patients over 18 years of age who have a clear diagnosis of insomnia. There will be no restrictions on sample size, treatment time, types of therapeutic drugs, race, culture, region, and gender.

#### 2.2.3 Interventions and comparators

The control group will be treated with conventional western medicine or placebo, and the treatment group will be treated with a single CPM or a single CPM plus conventional western medicine or placebo, which must be the same as the control group. There will be no restriction on dosage form, dosage and usage of CPM.

#### 2.2.4 Outcomes

The primary outcome indicator will be clinical efficacy, and secondary outcome indicators will include Pittsburgh Sleep Quality Index points, TDL Quality of Life Assessment Scale points, and adverse eventss.

### 2.3 Exclusion criteria

The efficacy evaluation does not meet the relevant standards; literature of non-RCTs, incomplete data, reviews, case experience reports, animal and cell experiments; patients with severe heart, lung, liver and kidney dysfunction, mental disorders, and malignant tumors.

### 2.4 Search strategy

We will search CNKI, Wanfang, VIP, CBM, Pubmed, Cochrane Library, Embase for the (RCTs of CPM in the treatment of insomnia, and then screen the relevant documents published at home and abroad. The limited publication time is from the establishment of the database to December 31, 2020. According to different database conditions, the subject words, key words and free words will be comprehensively searched to ensure the systematicness and integrity of the search. Taking PubMed as an example, the search strategy is shown in Table 1.

**Table 1:** Search strategy for PubMed.

#1	“Sleep initiation and maintenance disorders” [MeSH]
#2	“Disorders of initiating and maintaining sleep” [title/abstract] or “DIMS (disorders of initiating and maintaining sleep)” [title/abstract] or “early awakening” [title/abstract] or “awakening, early” [title/abstract] or “nonorganic insomnia” [title/abstract] or “insomnia, nonorganic” [title/abstract] or “primary insomnia” [title/abstract] or “insomnia, primary” [title/abstract] or “transient insomnia” [title/abstract] or “insomnia, transient” [title/abstract] or “rebound insomnia” [title/abstract] or “insomnia, rebound” [title/abstract] or “secondary insomnia” [title/abstract] or “insomnia, secondary” [title/abstract] or “sleep initiation dysfunction” [title/abstract] or “dysfunction, sleep initiation” [title/abstract] or “dysfunctions, sleep initiation” [title/abstract] or “sleep initiation dysfunctions” [title/abstract] or “sleeplessness” [title/abstract] or “Insomnia disorder” [title/abstract] or “insomnia disorders” [title/abstract] or “insomnia disorders” [title/abstract] or “insomnia” [title/abstract] or “insomnias” [title/abstract] or “chronic insomnia” [title/abstract] or “insomnia, chronic” [title/abstract] or “psychophysiological insomnia” [title/abstract] or “insomnia, psychophysiological” [title/abstract]
#3	#1 or #2
#4	“Medicine, Chinese traditional”[MeSH]
#5	“Traditional Chinese medicine” [title/abstract] or “Chung I Hsueh” [title/abstract] or “Hsueh, Chung I” [title/abstract] or “traditional medicine, Chinese” [title/abstract] or “Zhong Yi Xue” [title/abstract] or “Chinese traditional medicine” [title/abstract] or “Chinese medicine, traditional” [title/abstract] or “traditional tongue diagnosis” [title/abstract] or “tongue diagnoses, traditional” [title/abstract] or “tongue diagnosis, traditional” [title/abstract] or “traditional tongue diagnoses” [title/abstract] or “traditional tongue assessment” [title/abstract] or “tongue assessment, traditional” [title/abstract] or “traditional tongue assessments” [title/abstract]
#6	“Zaoren Anshen” [title/abstract] or “Shensong Yangxin” [title/abstract] or “Shugan Jieyu” [title/abstract] or “Shumian” [title/abstract] or “Baile Mian” [title/abstract] or “Danzhi Xiaoyao” [title/abstract] or “Liuwei Dihuang” [title/abstract] or “Danggui Liuhuang” [title/abstract] or “Zuogui” [title/abstract] or “Jiaotai” [title/abstract] or “Tianmeng” [title/abstract] or “Xuefu Zhuyu” [title/abstract] or “Anshen Jiannao” [title/abstract] or “Qiye Shenan” [title/abstract] or “Shunao Xin” [title/abstract] or “Anshen Buxin” [title/abstract] or “Bazhen” [title/abstract] or “Tianwang buxin” [title/abstract] or “Guipi” [title/abstract]
#7	#4 or #5 or #6
#8	“Placebo”[title/abstract]) or “randomized”[title/abstract]) or “Randomly”[title/abstract]) or “trial”[title/abstract]) or “groups”[title/abstract] or “randomized controlled trial”[title/abstract] or “controlled clinical trial”[title/abstract] or “randomized trial”[title/abstract]
#9	“Humans”[MeSH] or “homo sapiens”[title/abstract] or “man (taxonomy) “[title/abstract] or “man, modern”[title/abstract] or “modern man”[title/abstract] or “human”[title/abstract]
#10	#3 and #7 and #8 and #9

### 2.5 Study selection and data extraction

Two researchers will independently screen the literature, preliminarily screen the retrieval results by reading the topics and abstracts, and combining the inclusion and exclusion criteria, and then cross-check. In case of differences, 2 researchers will consult a third party to discuss and solve. The extracted data will include the first author, published year, sample size, basic information of patients, baseline situation, randomized method, intervention measures, treatment course, outcome index, etc.

### 2.6 Risk of bias assessment

The quality evaluation of each RCT will be independently evaluated by 2 researchers according to the improved Jadad scale.^[15]^ In case of disagreement, they will reach an agreement through negotiation or consult a third party to solve the problem. The evaluation contents will include: the generation of random sequence, the scheme of allocation and concealment, the implementation of blind method, withdrawal and missing visit. The total score of 1 to 2 is low quality, 3 to 4 is medium quality and 5 to 7 is high quality. In the evaluation process, if there is a lack of data, try to get it by contacting the author, and if it is impossible to get it, screen out the literature as appropriate.

### 2.7 Statistical analysis

We will use Revman 5.3 (Cochrane Collaboration, Copenhagen, Denmark), Stata 15.1 (Stata Corporation, College Station, TX) and ADDIS 1.16.8 software for NMA. The Binary variables will be expressed by odds ratio and its 95% confidence interval while the continuous variables will be expressed by mean difference or standardized mean difference and its 95% confidence interval. χ^2^ test will be used to analyze the heterogeneity and I^2^ to evaluate the heterogeneity. If I^2^ ≤ 50%, the heterogeneity is small, and the fixed effect model will used to combine the effect amount; if I^2^ > 50%, the heterogeneity is large, after excluding the influence of significant clinical heterogeneity, if the heterogeneity is small, the fixed effect model will be used; otherwise, subgroup analysis or sensitivity analysis will be used to deal with obvious clinical heterogeneity, eliminate heterogeneity factors or use random effects model to merge analysis. When there is a closed loop, the consistency of direct comparison and indirect comparison passes the consistency test. When the consistency test inconsistency factor is close to 0 or the hypothesis test odds ratio is close to 1, the direct and indirect evidences are considered to be consistent. The funnel diagram will be drawn to identify whether there is small sample effect evaluation. 4 Markov chain-Monte Carlo will be used to set the initial value. The number of initial update iterations of the model will be set to 50,000, and the number of continued update iterations will be set to 20,000. The first 50,000 will be used for annealing to eliminate the influence of the initial value. Sampling will start after 50,001. When the potential scale reduction factor tends to 1, the degree of convergence is satisfactory. We will draw the surface under cumulative ranking area to predict the order of efficacy.

The difference will be statistically significant with *P* < .05. In addition, “Stata - > metaninf” procedure will be used for sensitivity analysis: all the included studies will be excluded one by one to investigate the reliability of the research results; “Stata - > meta Fund / netfund” procedure will be used to draw an inverted funnel diagram to qualitatively evaluate the publication bias; “Stata - > metanbias” procedure will be used to conduct Begg rank correlation analysis and egger linear regression analysis to quantitatively evaluate publication bias.

### 2.8 Grading the quality of evidence

We will use the Grading of Recommendations Assessment, Development and Evaluation to evaluate the analysis results from 5 aspects: risk of bias, indirectness, inconsistency, imprecision, and publication bias. The outcome will be divided into 4 levels: high, moderate, low, and very low.

### 2.9 Ethics and dissemination

This study does not involve personal and human trial data and therefore does not require ethical approval.

## 3 Discussion

Insomnia is the most common sleep disorder in clinical practice. It refers to the continued difficulty of sleep initiation, reduced sleep events, damage to sleep integrity, or decreased sleep quality under adequate sleep opportunities and environments, and causes related daytime functional impairment. Daytime functional impairment includes fatigue, depression, agitation, physical discomfort, cognitive impairment, etc. Long-term chronic insomnia will reduce the patient's memory, reaction ability, judgment, and operational ability, which will not only affect social functions and reduce work efficiency, but also increase the risk of traffic accidents. Insomnia can also cause many physical discomforts such as dizziness, headache, muscle aches, palpitation, chest tightness, etc, which can increase the incidence of mental illness and cardiovascular disease. As social pressure and work rhythm continue to accelerate, it is expected that the proportion of patients with insomnia will further increase.

Although meta-analysis has proved that CPM is effective in treating insomnia.^[16,17]^ However, traditional meta-analysis can only perform pairwise comparisons, and there is no comparison and ranking of the efficacy of each drug. Therefore, we will use a NMA to rank various CPM to find the best therapeutic effect for insomnia. As far as we know, this is the first time that NMA has been used as a CPM to treat insomnia. We expect that through our research, clinicians and patients will pay attention to help in the treatment of insomnia.

## Author contributions

**Conceptualization:** Ruoyu Chao.

**Formal analysis:** Chunli Wu.

**Funding acquisition:** Jianlin Wu.

**Methodology:** Hongqiang An.

**Software:** Bing Li.

**Writing – original draft:** Ruoyu Chao.

**Writing – review & editing:** Jianlin Wu.
